# Shielding Gas Model for Annular Laser Metal Deposition of Reactive Materials in an Open Environment

**DOI:** 10.3390/ma19132874

**Published:** 2026-07-05

**Authors:** Bin Li, Jinchao Zhang, Sen Gu, Boyong Su, Jincan Cui, Wei Guo, Heng Wang

**Affiliations:** 1 School of Mechanical Engineering, Nantong Institute of Technology, Nantong 226002, China; libin19@ntit.edu.cn (B.L.);; 2 Jiangsu Key Laboratory of 3D Printing Equipment and Application Technology, Nantong Institute of Technology, Nantong 226002, China; 3 School of Optoelectronic Science and Engineering, Soochow University, Suzhou 215021, China; 4 School of Mechanical Engineering and Transit & School of Intelligent Manufacturing, Changzhou University, Changzhou 213164, China

**Keywords:** annular laser metal deposition (ALMD), reactive materials, CFD, CCD, open environment

## Abstract

A primary challenge in successfully manufacturing reactive materials for laser metal deposition (LMD) is to prevent oxidization. To address the oxidation susceptibility of titanium alloys in open environments, a local atmosphere protection model was developed. Using Ansys Fluent 2023R1 software, the effective protective atmosphere range provided by the local shielding device under varying shielding gas flow rates was investigated in detail, and a mathematical model for the effective protection area was obtained through quadratic polynomial fitting. A quadratic regression model linking process parameters with the length of high-temperature zones was established using central composite design (CCD). By integrating these two models, a local shielding gas model for LMD titanium alloys in an open environment was formulated. Validation experiments demonstrated excellent morphology of the single layers with shiny silver. The local shielding atmosphere can effectively protect the high-temperature region. These findings provide a basis for the deposition of active materials in an open environment and the selection of appropriate shielding gas flow rates.

## 1. Introduction

Laser metal deposition (LMD) has received widespread attention due to its ability to produce near-net-shape components or repair parts [1,2]. Through the interaction of the laser and material, custom-shaped parts, functionally graded materials and multi-material structures can be manufactured [3,4]. Titanium alloys, such as Ti6Al4V, have been applied in various fields including aerospace, biomedical, petrochemical and marine industries. They have many advantages, such as outstanding specific strength, superior corrosion resistance and excellent biocompatibility [5]. Nowadays, the LMD technology for depositing titanium alloy components has garnered widespread attention.

The titanium alloys manufactured by LMD undergo vigorous reactions with oxygen under high-temperature conditions. Impurity content in deposition samples significantly affects the properties of deposited alloys. Material performance will deteriorate due to atmospheric contamination, such as chemical composition, surface topography, microstructure, and mechanical properties [6,7,8]. Chen et al. [9] found that high oxygen concentrations caused oxidation of Ti6Al4V welds, resulting in purple and blue discoloration. According to the American Welding Society (AWS), protective measures must be taken to prevent oxidation of titanium alloys when the temperature exceeds 400 °C in the atmosphere [10]. Yang et al. [11] demonstrated that oxidation affected the shape of molten pools, and low oxygen concentrations decreased scum on the surface of molten pools. This fully demonstrates the importance of controlling oxygen pollution. Bermingham et al. [12] investigated the oxidation sensitivity of deposited Ti6Al4V components, and pointed out that high oxygen concentrations can form a thick and stable oxide layer. The microhardness near the surface was higher. Overall, LMD of reactive materials needs a closed inert gas chamber, which can maintain a low oxygen concentration through argon purification treatment [13]. However, it is not suitable for manufacturing large components or in situ repair parts in a closed chamber.

An open environment with a local inert shield is more conducive to free-form forming processes, enabling the formation of large-sized and complex-shaped parts [14]. In addition, the process cost declines due to the decrease in gas consumption. The shielding nozzle designed with a special structure can directly inject protective gas into the processing region, and a low-oxygen atmosphere is created around the processing zone to prevent oxidation [15]. Thus, the parts can be formed in an open environment. Ding et al. [16] evaluated the shielding performance of the designed shielding nozzle based on computational fluid dynamics (CFD). Bernauer et al. [17] developed a shielding nozzle for the LMD process based on CFD. The tested experiments validated its effectiveness. However, these studies did not provide a quantitative assessment of shielding performance. Yang et al. [11] investigated the effect of shielding gas flow rates on forming quality. They pointed out that an increase in the shielding gas flow rate expanded the area of low-oxygen atmosphere and facilitated the expansion of the molten pool. However, a model for low-oxygen atmosphere regions has not been established. Ruiz et al. [14] developed an asymmetric shielding nozzle. The feasibility experiment of depositing samples in an open environment was verified. However, the principles for selecting the required shielding gas flow rate under different process parameters are not yet clear in an open environment.

During the LMD process, the protective performance of the shielding nozzle is closely dependent on the flow rate of the shielding gas [18,19]. When the flow rate of the shielding gas is insufficient, the local inert atmosphere fails to fully cover the high-temperature zones. Oxidation reactions occur in areas with poor protection. When the shielding gas flow rate is excessively high, the inert gas will cause serious waste. In addition, it increases the gas velocity at the nozzle outlet, which reduces the stability of the melt pool and deposition quality. Furthermore, the high-temperature areas that require inert gas protection are affected by process parameters. Therefore, appropriate shielding gas consumption must be determined for different process parameters. It is crucial to build a shielding gas model for the manufacture of reactive materials in open environments.

In this study, a shielding gas model for ALMD of Ti6Al4V alloy in an open environment is investigated. The effective protection area provided by the shielding device is simulated using CFD, and its model is established using quadratic polynomial fitting. Then, the regression model for the length of high-temperature area behind the melt pool is established based on ANSYS 2023R1 and central composite design (CCD). Finally, a local shielding gas model is obtained through simultaneous analysis of above two models. It can provide a basis for determining the optimal shielding gas consumption for LMD of titanium alloys in open environments.

## 2. Experimental Procedure

### 2.1. ALMD System and Local Shielding Nozzle Design

The ALMD system consisted of a IPG fiber laser (IPG Photonics, Oxford, MA, USA) with a maximum power of 2 kW, a six-axis KUKA mobile robot (TIE Industrial, La Vergne, TN, USA), and a GTV powder feeder (Luckenbach, Germany) [20]. The annular laser head was self-designed, and the annular laser can be received through an optical path conversion [21]. The powder feeding tube was located at the center of the annular laser beam. A self-made double-layer shielding nozzle was used to prevent oxidation, and installed coaxially with the powder feeding nozzle. The central powder feeding tube sprays powder vertically with a small divergence angle. Therefore, the outer gas flow from the shielding nozzle has a relatively small impact on the powder stream. The shielding nozzle can provide a local inert atmosphere with a low oxygen level for the molten pool, thereby replacing the traditional sealed chamber. More details about the annular beam powder feeding nozzle and the double-layer shielding device are available in reference [15].

### 2.2. Definition of Effective Local Atmosphere Protection

**Figure 1:**
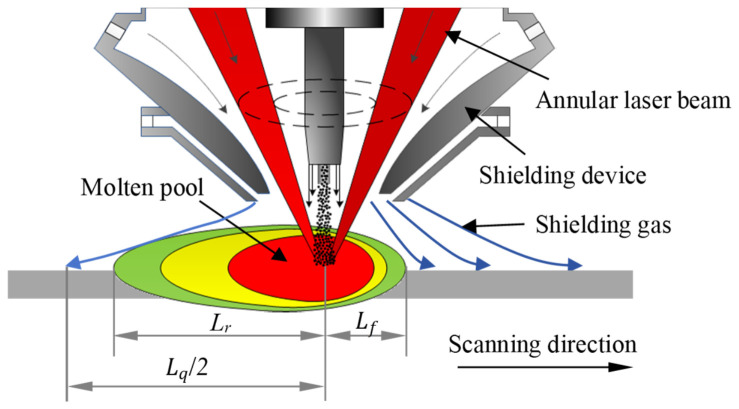
Schematic diagram of high-temperature area of melt pool and effective protection area.

In an open environment, the oxidation of deposited active materials depended on the competitive relationship between the effective protection area generated by the shielding nozzle and the high-temperature area of the melt pool. From Figure 1, it can be seen that when the high-temperature region was entirely covered by the effective protection area, the oxidation phenomenon can be suppressed. Titanium undergoes intense chemical reactions with oxygen when the temperature exceeds 400 °C. Therefore, the region above 400 °C (marked in green) was defined as the high-temperature zone of the melt pool. It can be observed that the temperature field of the melt pool exhibited a comet-like distribution, and the length of the high-temperature region, i.e., scanning direction, is significantly greater than its width. Therefore, only the length direction of the high-temperature region is investigated. Furthermore, the length of the high-temperature area behind the melt pool ($L_{r}$) is greater than that in front of it ($L_{f}$).

The effective protection area ($L_{q}$) formed by the shielding device is circumferentially uniform and coaxial with the laser beam. In addition, the $L_{r}$ is greater than both $L_{f}$ and the width of the melt pool. Consequently, when half of $L_{q}$ completely covers $L_{r}$, it indicates that the entire high-temperature area receives effective protection and the oxidation reaction is suppressed during the ALMD process, as shown in Figure 1. The effective protection zone and high-temperature zone of the melt pool were obtained through simulation.

### 2.3. CFD of the Shielding Nozzle

The protective performance of the shielding nozzle was studied using a three-dimensional (3D) CFD model. Based on the path conforming method, a tetrahedral mesh was adopted. To investigate the gas flow patterns surrounding the inlets, a small grid was created near the inlets. The k-epsilon turbulence model and the SIMPLE algorithm method were employed to study the oxygen concentration distribution. Pure argon with a purity of 99.99% was used for all gases. Each argon inlet was set as a velocity inlet, while the air domain was set as a pressure outlet. Velocity inlets were set at a constant value, and each inlet velocity was calculated based on gas flow rate and inlet diameter. The pressure at the boundary was atmospheric pressure, since the emerging gas was surrounded by the atmosphere. The composition was nitrogen (78.08%), oxygen (20.95%) and argon (0.97%) by volume.

A low-oxygen atmosphere in the deposition region is crucial for preventing oxidation during the processing. Ding et al. [16] explored the influence of oxygen contents in a closed chamber on the performance of titanium wire + arc additive manufacture (WAAM) samples. They pointed out that 2000 ppm was selected as the highest acceptable oxygen content to avoid oxidation. In the present work, the effective protective area beneath the shielding nozzle was taken to be the zone whose oxygen content was lower than 0.2%. To develop a mathematical model for the effective protection area $L_{q}$, CFD simulations were used to analyze $L_{q}$ values under various shielding gas flow conditions. The shielding gas flow rate was limited to a range of 18–50 L/min. Detailed shielding gas flow rates are given in Table 1.

### 2.4. Calculation of Temperature Field

$L_{r}$ was evaluated by the temperature field using ANSYS. In order to enhance computational accuracy, meshes with different dimensions were applied in different regions [22]. The mesh dimensions of the substrate were 0.2 mm × 0.2 mm × 0.5 mm, while those of the deposited layer were 0.2 mm × 0.2 mm × 0.16 mm. The mesh dimensions from the deposited layer region toward the substrate edge gradually increased.

The laser deposition process involves nonlinear transient thermal analysis. Therefore, solid70 element was selected for meshing. This element has eight nodes, which enhanced the computational efficiency. Each node possesses merely one degree of freedom for temperature and can conduct heat in three directions, making it suitable for three-dimensional transient thermal analysis. Heat convection is represented by the convective coefficient. The heat distribution of the annular laser is expressed by an exponential decay body heat source model, as seen in Equation (1). The nonlinear equations are calculated by the Newton–Raphson method [23]:

(1) $$I(x,y,z)=\frac{2P\eta }{\pi (R^{2}\;-\;r^{2})h}\exp\left[-8\frac{{\left(\sqrt{x^{2}\;+\;y^{2}}\;-\;\frac{R\;+\;r}{2}\right)}^{2}}{(R\;-\;r{)}^{2}}\right]\cdot \exp\left[-\frac{\left|z\right|}{h}\right]$$

where P is the laser power, η is the absorption coefficient of materials to laser energy, h is the laser penetration depth, R is the external diameter of the annular laser, r is the inner diameter of the annular laser, and x, y and z represent the position of the laser-heating center point in the three-coordinate system.

$L_{r}$ is related to heat input, while ALMD parameters affect heat input. Two ALMD parameters, namely laser power (*P*) and scanning speed (*V*), were selected as input variables, and $L_{r}$ was considered as an output response. An increase in *P* or a decrease in *V* increases the heat input, resulting in an increase in $L_{r}$. Table 2 presents the suitable ranges for two chosen parameters at five levels. The $L_{r}$ model was established based on the central composite design (CCD) approach. Table 3 shows a 13-run CCD matrix, and it consists of 8 axial points and 5 center points.

## 3. Results and Discussion

### 3.1. The Effective Protection Area of Shielding Device

#### 3.1.1. CFD Modeling Results

**Figure 2:**
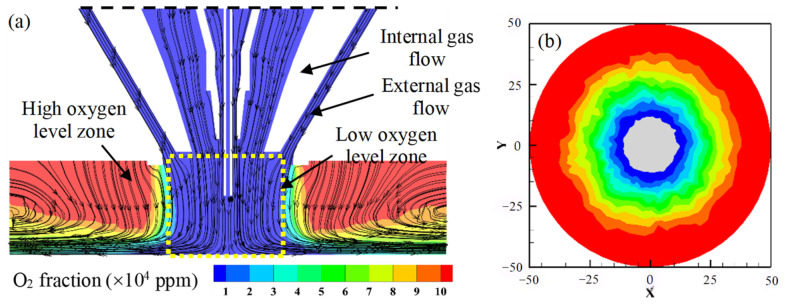
Oxygen content distribution: (**a**) longitudinal section; (**b**) transverse section.

Taking No.5 in Table 1 as an example, the oxygen content distribution at different sections is shown in Figure 2. Figure 2a shows the oxygen content distribution and gas streamline in the longitudinal section. Beneath the shielding nozzle, a jet of nearly cylindrical shape indicated in blue was produced. In this zone, the airflow is distributed in parallel without generating turbulence or upward flow, and the oxygen content is at a relatively low level. In addition, the shielding gas flow produces a laminar layer of a certain thickness on the substrate surface, which can protect the melt pool from the invasion of external air.

**Figure 3:**
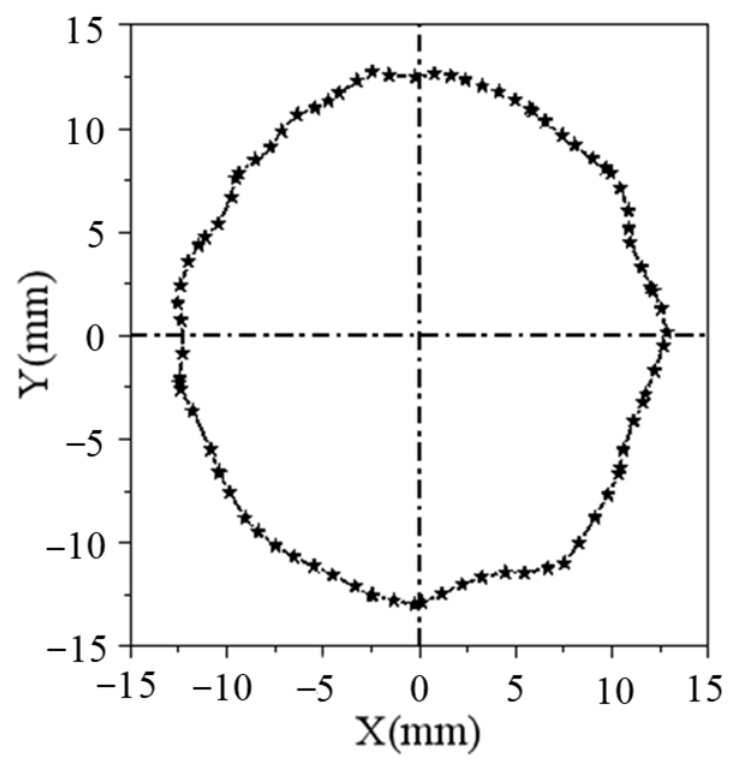
Outer contour of oxygen content below 2000 ppm.

Figure 2b shows the oxygen content distribution in the cross-section of the substrate surface. Regions with oxygen content below 2000 ppm are marked in gray, indicating that these areas can prevent oxidation of the melt pool. Figure 3 shows the external contour line of the gray region extracted from Figure 2b, and the protection area ($L_{q}$) can be calculated by using Equation (2). From Figure 3, it is observed that the effective protection area is approximately circular, and it can prevent the high-temperature region from contamination by contact with air. In this case, the $L_{q}$ is 25.28 mm.

(2) $$L_{q}=2\sum_{i=1}^{N}\sqrt{{x_{i}}^{2}+{y_{i}}^{2}}/N$$

where $x_{i}$ and $y_{i}$ denote the coordinates of each contour point, and *N* represents the total count of contour points.

#### 3.1.2. Model of $L_{q}$

**Figure 4:**
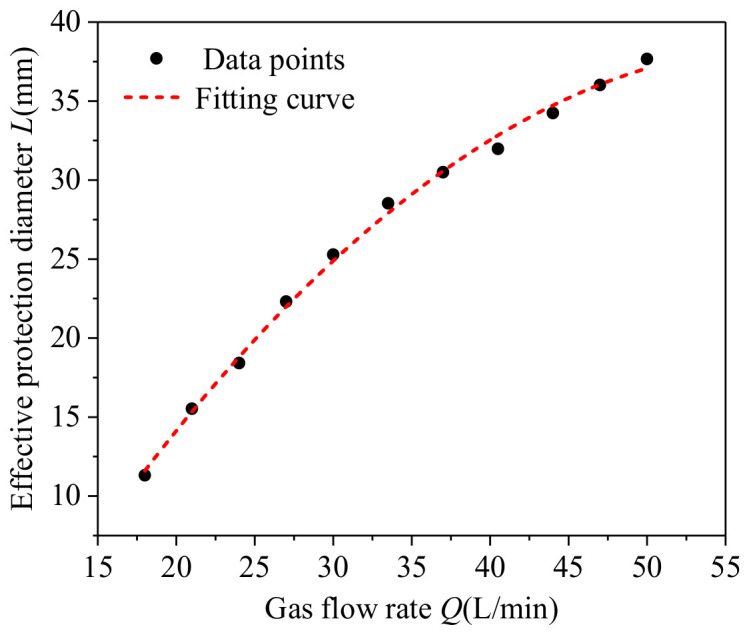
The fitting curve of $L_{q}$.

$L_{q}$ values are obtained by CFD simulation and Equation (2) at different shielding gas flow rates, and the fitting curve of $L_{q}$ using a quadratic polynomial is shown in Figure 4. Obviously, the fitting curve (indicated by red dotted line) closely matches the data point trend. The finally obtained quadric polynomial regression model for $L_{q}$ is shown in Equation (3):

(3) $$L_{q}=-16.6856+1.8509Q-0.0155Q^{2}$$

where *Q* is the flow rate of the shielding gas.

**Figure 5:**
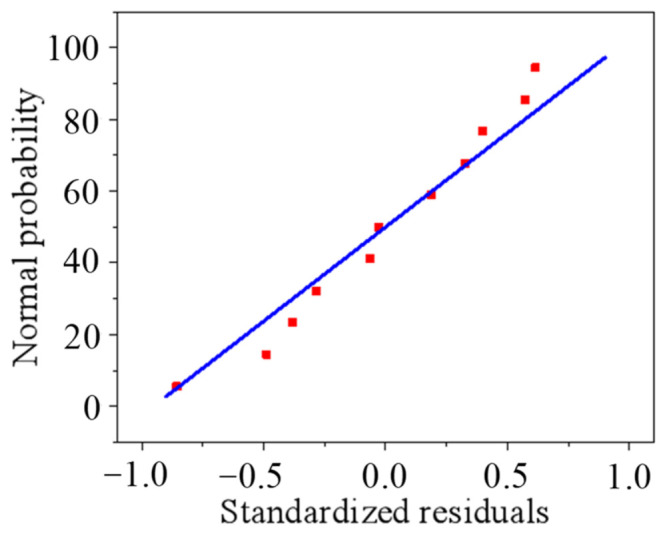
Plot of normal probability of residuals.

**Figure 6:**
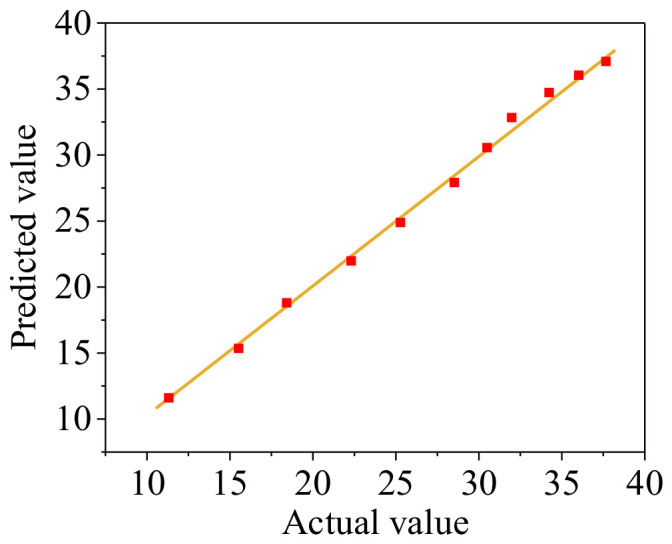
Plot of predicted versus actual value.

The significance of the $L_{q}$ model was assessed by analysis of variance (ANOVA). In Table 4, the *p*-value is less than 0.05, which confirms the statistical significance of the regression model. Furthermore, the coefficient of determination *R*^2^ and adjusted *R*^2^ are near 1, demonstrating that the model exhibits good adequacy and fit quality. Figure 5 shows the normal probability plot of residuals. The residuals present an approximate linear trend, proving that the residuals follow a normal distribution. The adequacy of $L_{q}$ model is estimated by comparing actual and predicted values, as shown in Figure 6. The predicted values closely match the actual values, indicating that the generated model has good predictive performance.

### 3.2. The High-Temperature Region of ALMD Process

#### 3.2.1. Analysis of the Temperature Field

**Figure 7:**
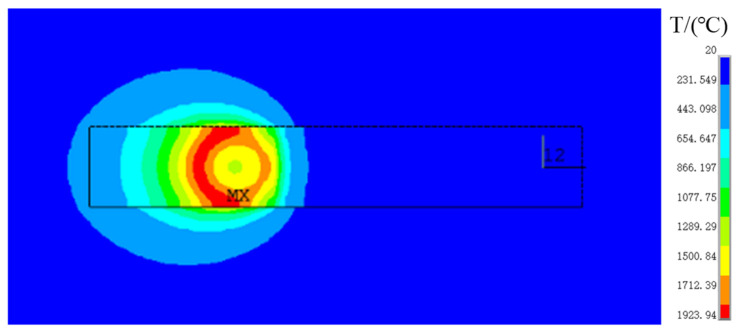
Temperature field of melt pool.

Take one of the cases (No.1) in Table 3 as an example. Figure 7 shows the temperature field of the molten pool during the ALMD process. The temperature distribution of the molten pool exhibits a comet-like trailing pattern. The temperature gradient ahead of the melt pool exceeds that at its rear. This is mainly because the front of the melt pool is subjected to high heat input from the laser, causing a rapid increase in temperature and thus forming a steep temperature gradient. At the rear edge of the melt pool, heat dissipates into the surrounding base metal and air, while the heating effect of the laser diminishes, resulting in a reduced temperature gradient. Unlike the typical solid Gaussian spot characterized by high temperature at the center and low temperature in the surrounding region, the temperature distribution of the annular laser exhibits higher temperature in the surrounding region and lower temperature at the center. In addition, the temperature at the rear of the melt pool is higher than at its front.

**Figure 8:**
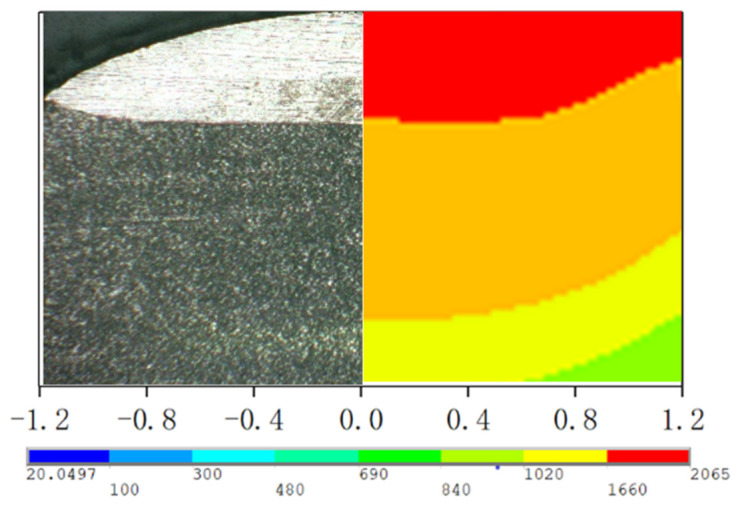
Comparison chart between simulation and experimental results of the molten pool.

Figure 8 compares the simulation result of the cross-section of single layer with the experimental result. According to the melt pool boundary criterion, the model is considered valid when the simulated solid–liquid interface aligns with the boundary of the substrate remelting zone. The boundary temperature of the remelting zone was set to 1660 °C. The simulated melt pool shape fundamentally matches the experimental one, which verifies the reliability of the temperature field model.

#### 3.2.2. Establishment of $L_{r}$ Model and ANOVA

As shown in Table 3, the length of the high-temperature zone at the rear of the melt pool ($L_{r}$) varies with different combinations of parameters. CCD is used to relate the two chosen parameters to the $L_{r}$ response. Based on the least squares method, the developed quadric polynomial regression for $L_{r}$ is shown in Equation (4):

(4) $$\begin{array}{cc} L_{r}=\; & 0.7869+7.67972\times 10^{-3}P-0.28512V+9.225\times 10^{-5}PV-\\ & 1.35625\times 10^{-7}P^{2}+0.021119V^{2} \end{array}$$

The ANOVA results are shown in Table 5. The obtained *p*-value is less than 0.05, confirming the statistical significance of the $L_{r}$ model and each contribution term. For $L_{r}$ model, significant terms include *P*, *V* and *V*^2^. Among the parameters, *P* has a larger effect than *V*. Furthermore, all the fit statistics, *R*^2^, adjusted *R*^2^ and predicted *R*^2^, are 1, which indicates good adequacy and fit quality of the $L_{r}$ model. The adequate precision (*A_P_*) value is well above 4, suggesting that the fit statistics are reasonably consistent [24,25].

**Figure 9:**
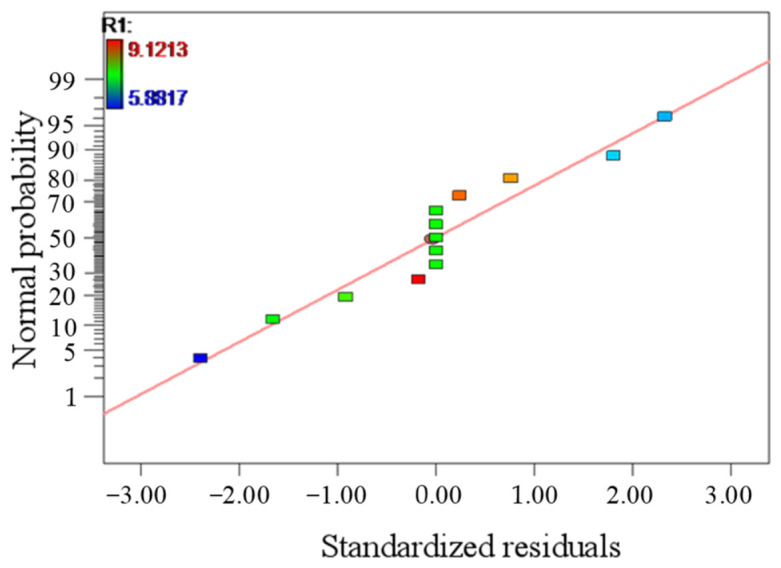
Plot of normal probability of residuals.

**Figure 10:**
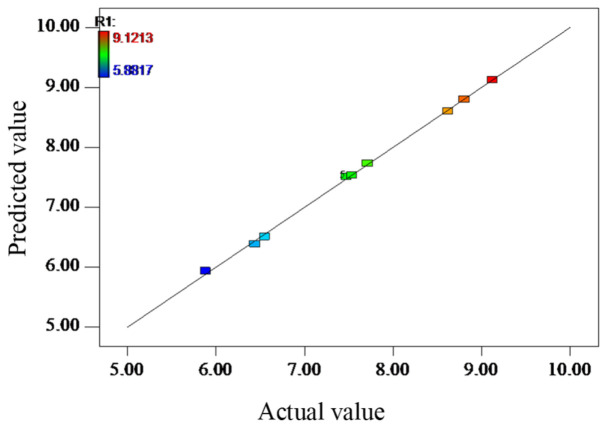
Plot of predicted versus actual value.

To evaluate the adequacy of the $L_{r}$ model, Figure 9 shows the normal probability plot of residuals. The plot indicates that the residuals exhibit an approximate linear trend, verifying that they are normally distributed. The adequacy of the $L_{r}$ model is further estimated by comparing actual and predicted values, as shown in Figure 10. The data points closely following the diagonal suggest a satisfactory fit of the developed model.

## 4. Local Shielding Gas Model

### 4.1. Establishment of the Model

When half of $L_{q}$ completely covers $L_{r}$, it indicates that the entire high-temperature area obtains effective inert protection during the ALMD process. The $L_{q}$ and $L_{r}$ values were obtained through simulation.

As can be seen from Section 2.2 and Section 3, to prevent oxidation of the deposited layer, different process parameters must be matched with appropriate shielding gas flow rates. When half of $L_{q}$ exceeds $L_{r}$, the ALMD process will not be contaminated by oxidation. Therefore, the local shielding gas flow rate *Q* obtained by $L_{q}/2=L_{r}$ can be regarded as the minimum shielding gas flow rate capable of effectively protecting the high-temperature area. Through combining Equations (3) and (4), the relationship model between the minimum shielding gas flow rate *Q*_min_ and process parameters is derived, as shown below:

(5) $$\frac{L_{q}}{2}=L_{r}$$

(6) $$\begin{array}{l} \left(-16.68558+1.8509Q-0.01551Q^{2}\right)/2=0.7869+7.67972\times 10^{-3}P-\\ 0.28512V+9.225\times 10^{-5}PV-1.35625\times 10^{-7}P^{2}+0.021119V^{2} \end{array}$$

(7) $$Q_{\min}=59.67-4.03\times 10^{-8}\varphi$$

(8) $$\begin{array}{c} \varphi \\ =\sqrt{1.08P^{2}-7.33PV-6.1\times 10^{14}P-1.68\times 10^{15}V^{2}+2.26\times 10^{16}V+1.47\times 10^{18}} \end{array}$$

### 4.2. Confirmation Testing

The reliability of the local shielding gas model primarily depends on the accuracy of the $L_{r}$ model. To verify the validity of developed model, confirmation experiments were conducted. Three sets of parameters were chosen to perform validation experiments, as shown in Table 6. *Q*_min_ was calculated using Equation (7), but the shielding gas flow rates were rounded during the experiments; this is because the adjustment accuracy of the gas flow meter used in the experiment is 1 L/min. As shown in Table 6, the deviation between the actual value and predicted value of $L_{r}$ is less than 4%, indicating that the model exhibits high reliability. In addition, the $L_{r}$ values of each set are less than $L_{q}/2$, which meets the requirements for effective protection in Section 2.2, indicating that the local inert atmosphere can provide good protection for high-temperature areas.

**Figure 11:**
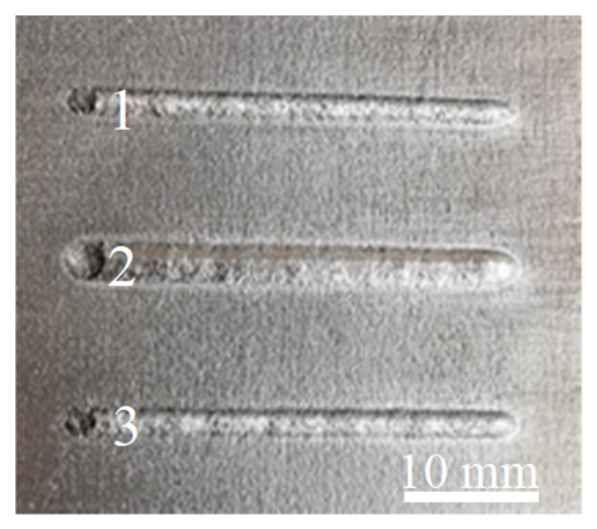
Validated layers with shiny silver.

A single layer of Ti6Al4V titanium alloy was deposited under parameters given in Table 6, and the morphology of deposited layers is shown in Figure 11. It can be observed that all deposited layers exhibit smooth and shiny silver. This indicates that the local inert atmosphere generated by the calculated shielding gas flow rate can effectively shield the high-temperature area, and oxidation contamination is suppressed. Verification tests confirm that the *Q*_min_ model exhibits high reliability, and it can be used for calculating shielding gas flow rates under different ALMD processes.

## 5. Conclusions

An ALMD process method for reactive material in an open environment was proposed. The local shielding gas model was established to obtain an oxidation-free process. The main findings are follows:

(1) A statistical model for the effective protection area is established based on CFD simulation and quadratic polynomial fitting. The predicted values closely match the experimental ones.
(2) A statistical model for the length of the high-temperature area at the rear of the melt pool is established based on ANSYS and CCD. The model exhibits good adequacy.
(3) A local shielding gas model for ALMD of reactive material in an open environment is obtained. Validation experiments demonstrate that the local inert atmosphere generated by the calculated shielding gas model can effectively shield the high-temperature area, and the deposited layers exhibit smooth and shiny silver. This breaks through the limitations of deposition of reactive materials in a closed inert gas chamber.

## Figures and Tables

**Table 1:** The different flow rates of shielding gas for CFD simulation.

No.	Flow Rates of Shielding Gas/(L·min^−1^)		
	Total Gas	Internal Gas	External Gas
1	18	14	4
2	21	15	6
3	24	16	8
4	27	17	10
5	30	18	12
6	33.5	21	12.5
7	37	24	13
8	40.5	27	13.5
9	44	30	14
10	47	32.5	14.5
11	50	35	15

**Table 2:** Levels of selected process parameters.

ALMD Parameters	Units	Levels				
		−2	−1	0	+1	+2
Laser power (*P*)	W	750	808.58	950	1091.42	1150
Scanning speed (*V*)	mm/s	4	4.59	6	7.41	8

**Table 3:** Settings of CCD process variables and corresponding measurements.

No.	ALMD Parameters		Responses
	*P* (W)	*V* (mm/s)	*L_r_*
1	950	6	7.5356
2	950	8	7.7115
3	950	6	7.5356
4	1091.42	4.59	8.6225
5	750	6	5.8817
6	950	6	7.5356
7	1091.42	7.41	8.8060
8	808.58	7.41	6.5482
9	1150	6	9.1213
10	950	4	7.4713
11	950	6	7.5356
12	950	6	7.5356
13	808.58	4.59	6.4385

**Table 4:** ANOVA for the fitted model of $L_{q}$.

	Df	Sum of Squares	Mean Square	F-Value	*p*-Value
Model	2	758.11831	379.05915	1370.20818	7.17844 × 10^−11^
Residual	8	2.21315	0.27664		
Cor total	10	760.33145			
R^2^ = 0.99709 R^2^_Adj_ = 0.99636					

**Table 5:** ANOVA for the fitted model of $L_{r}$.

Source	Sum of Squares	Df	Mean Square	F-Value	*p*-Value	
Model	10.24	5	2.05	1543.88	<0.0001	Significant
*P*	10.18	1	10.18	7670.94	<0.0001	
*V*	0.050	1	0.050	37.74	0.0005	
*PV*	1.362 × 10^−3^	1	1.362 × 10^−3^	1.03	0.3448	
*P* ^2^	5.118 × 10^−5^	1	5.118 × 10^−5^	0.039	0.8499	
*V* ^2^	0.012	1	0.012	9.35	0.0184	
Residual	9.287 × 10^−3^	7	1.327 × 10^−3^			
Lack of fit	9.287 × 10^−3^	3	3.096 × 10^−3^			
Pure error	0	4	0			
Cor total	10.25	12				

*R*^2^ = 0.9991, *R_Adj_*^2^ = 0.9984, *R_pred_*^2^ = 0.9936, *A_P_* = 128.920.

**Table 6:** Process parameters for the validation test.

No.	Process Parameters		Shielding Gas Flow Rate *Q*_min_*/*(L/min)		$L_{q}$/2 (mm)	$L_{r}$		Error/%
	*P*/W	*V*/(mm/s)	Calculated Value	Adjusted Value		Predicted Value	Actual Value	
1	830	5.2	19.18	20	7.07	6.55	6.34	3.31
2	1050	6	22.12	23	8.84	8.33	8.43	−1.12
3	750	4	18.22	19	6.44	5.94	5.82	2.06

## Data Availability

The original contributions presented in this study are included in the article. Further inquiries can be directed to the corresponding author.
